# Alcohol consumption as a socially contagious phenomenon in the Framingham Heart Study social network

**DOI:** 10.1038/s41598-024-54155-0

**Published:** 2024-02-24

**Authors:** Maarten W. J. van den Ende, Han L. J. van der Maas, Sacha Epskamp, Mike H. Lees

**Affiliations:** 1 Psychological Methods, University of Amsterdam, Amsterdam, 1001 NK The Netherlands; 2 Institute of Advanced Studies, University of Amsterdam, Amsterdam, 1012 GC The Netherlands; 3 Department of Psychology, National University of Singapore, Singapore, 117570 Singapore

**Keywords:** Human behaviour, Drug regulation, Public health, Psychology and behaviour

## Abstract

We use longitudinal social network data from the Framingham Heart Study to examine the extent to which alcohol consumption is influenced by the network structure. We assess the spread of alcohol use in a three-state SIS-type model, classifying individuals as abstainers, moderate drinkers, and heavy drinkers. We find that the use of three-states improves on the more canonical two-state classification, as the data show that all three states are highly stable and have different social dynamics. We show that when modelling the spread of alcohol use, it is important to model the topology of social interactions by incorporating the network structure. The population is not homogeneously mixed, and clustering is high with abstainers and heavy drinkers. We find that both abstainers and heavy drinkers have a strong influence on their social environment; for every heavy drinker and abstainer connection, the probability of a moderate drinker adopting their drinking behaviour increases by $40\%$ and $18\%$, respectively. We also find that abstinent connections have a significant positive effect on heavy drinkers quitting drinking. Using simulations, we find that while both are effective, increasing the influence of abstainers appears to be the more effective intervention compared to reducing the influence of heavy drinkers.

## Introduction

Alcohol dependence is the result of a complex interaction between many factors: social factors, from general life satisfaction to availability of the substance; psychological factors, such as choice processes and craving; and genetic vulnerabilities. Although extensive research has focused on the impact of the social environment on alcohol dependence^1,2^, the underlying interactions are still recognized as complex and multifaceted^3,4^. Initial use is affected by parental influence and exposure to peers who use drugs^5^. Peer pressure can facilitate abusive behaviour^6^, while social norms and stigma may make it difficult to seek help^3^. On the other hand, social support can also be crucial in recovery from alcohol abuse: community support is a key element in many recovery programs^7^. While there are many psychological theories and formal models of alcohol use, the impact of the social environment is often ignored^8^. At the same time, social approaches often forgo the exact structure of the social environment as the exact process of contagion of alcohol use is not well understood. However, in recent studies, the significance of network structure in contagion processes has increasingly been acknowledged. This has become particularly apparent during the COVID-19 pandemic, where there is a growing demand for modelling efforts that incorporate network structure^9,10^. Similarly, in the context of alcohol use, the importance of accounting for population heterogeneity has been demonstrated^8,11,12^. Here, we leverage longitudinal social network data from the Framingham Heart Study to explore the influence of the structure of the social environment on alcohol consumption patterns.

The influence of social connections on behaviours such as alcohol consumption, eating habits, depression, sleep patterns and smoking has been compared to the spread of infectious diseases^13–15^. The concept of ‘social contagion’ captures this phenomenon and suggests that mathematical models commonly used in epidemiology may be well suited to unravelling the dynamics of the spread of such behaviours. While numerous studies have studied the social transmission of different behaviours^16,17^, the application of epidemiological frameworks to noncommunicable diseases is still in its infancy. Methodological innovations have emerged that incorporate factors such as group interactions, context-dependent relationships and multiplex networks^18–20^. Social contagious models have primarily examined obesity, smoking, and information dissemination^21–23^, often relying on theoretical network structures rather than empirical data. By analysing network data from the Framingham Heart Study^24,25^, Hill and colleagues stand out in this area. Our current study follows their approach by using an epidemiological model to investigate how alcohol use spreads through social networks.

Furthermore, the binary classification of individuals as drinkers or non-drinkers may not accurately reflect the spectrum of alcohol use observed, as a large proportion of the population consumes alcohol in a relatively controlled and unproblematic manner. To address this, a three-tiered classification system - abstainers, moderate drinkers and heavy drinkers - has been proposed^26^. The current study aims to assess the implications of this nuanced categorisation and its effectiveness in providing a more intricate understanding of drinking patterns. We also assess the consistency of the observed data with the assumptions of our epidemiological model and identify significant parameters that inform us about the mechanisms behind the transmission of drinking behaviour. Finally, we aim to dissect the complexity of the spread of alcohol consumption by analysing the role of different drinking categories. Understanding the extent of social influence exerted by each category and their respective vulnerabilities is crucial. In addition, simulation experiments designed to test potential public health interventions will allow us to assess the effectiveness of different strategies aimed at mitigating the spread of alcohol use.

## Background

### Infectious disease modelling

Infectious disease models have been used extensively to model and predict epidemics in large populations. These models describe the dynamics of well-mixed subpopulations (e.g. susceptible, infected, recovered) as sets of ordinary differential equations. For some populations, the assumption of homogeneous mixing holds, and these models, although simple, accurately represent the dynamics of real-world spread^27,28^ and do not require social structure or more complex spread dynamics. These models compartmentalise individuals based solely on their physiological state: they are either ‘susceptible’ (S) to the disease or ‘infected’ (I) once they have contracted the disease. If immunity is acquired, they switch to the ‘recovered’ (R) state (SIR model). Or, if they can become infected again after recovery, they return to the ‘susceptible’ population (SIS model).

In these models, there are two main parameters that describe the behaviour of the disease: the rate at which infected individuals can spread the disease to susceptible individuals, and a constant rate of recovery. The reproduction number $R_0$, defined as the infection rate divided by the recovery rate, can already give a good indication of the infectiousness and the future course of the disease^29^. The simplicity of these models allows them to be solved analytically, which helps us to better understand their dynamics. As such, they can help policy makers make accurate predictions and explore scenarios of disease spread, such as the recent Covid19 outbreak^10,30^.

### Social contagion of alcohol use

Although alcohol consumption has been shown to behave like a ‘socially contagious’ behaviour^14,15^, it differs from infectious diseases and other behaviours in a number of ways that affect the precise modelling approach. First, one should apply an SIS-type model rather than an SIR-type model, as it is impossible to become immune when dealing with behaviour. Secondly, it has been shown that there is a large, stable, common moderate drinking state in which $98\%$ of years of recreational use are followed by another year of moderate drinking^26^. This moderate drinking consists of an average weekly consumption of one to seven drinks for women and one to fourteen drinks for men^31^. When trying to understand longitudinal patterns, it may therefore be important to distinguish moderate or recreational drinking from heavy drinking, which is associated with mental and biophysical health risks. We show that it is important to distinguish between abstainers, moderate drinkers and heavy drinkers, not only in terms of biophysiological consequences, but also to capture the dynamics of their spread across a population. Finally, while infectious diseases can generally only be transmitted through physical contact with an infected person, behaviour can also be adopted through a variety of other factors. Examples include cultural changes such as changes in normality^32,33^, differences in availability, advertising that promotes or discourages alcohol use, and the effects of policy interventions. This non-social or ‘spontaneous’ or ‘automatic’ transition must therefore be taken into account. This applies not only to increases in drinking, but also to reductions and cessation.

However, within the field of epidemiology, there is growing evidence that the properties of real-world social topology resulting from the heterogeneous connectivity patterns have an irrefutable impact on the behaviour of epidemic spread^9,28,34^, and the inclusion of explicit representations of these structures has been advocated since early 2000^35^. While some diseases can spread simply by individuals being in the same environment, behaviours that spread socially do so slowly, mostly through individuals with whom one has social ties and spends a lot of time. As there is great heterogeneity in these social ties^36^, describing the social environment of individuals becomes even more important for representing the interpersonal spread of behaviour.

A basic implementation of social structure in SIR-type models is to increase the number of compartments, for example by grouping into different age or risk groups, or by compartmentalising spatially^35^. Similar approaches have been applied to noncommunicable diseases^8,28^. For example, in the modelling of university binge drinking by^30^, where individuals from each starting year are separated into different spatial compartments. Homogeneous mixing still occurs within individuals from these years, but mixing, and hence contagion, between individuals from different years is reduced. However, a more accurate representation of social structure is provided by social networks^34^, where each individual (node) has connections (edges) to other individuals with whom they have a social relationship^37–41^. Constraining the model to a social network thus implies that an infected individual can only spread their behaviour to others with whom they are socially connected.

### Epidemiological models on networks

Modelling infectious processes in a social network has significant implications, as disease transmission no longer depends solely on epidemiological parameters, but also on the properties of the network. The added complexity of the network structure makes it implausible to solve the dynamics analytically without making simplifications to the network structure or relying on approximations such as the mean-field pairwise approximation^25,28^. However, these simplifications may only be reliable if social spread is significantly lower than the rate of spontaneous transitions. Therefore, simulation studies are the most reliable and preferred method to explore social dynamics.

Network connectivity plays a critical role in disease transmission; a highly connected network facilitates rapid spread, while a sparsely connected network can significantly slow disease transmission^42^. The degree of clustering is also relevant; if the network consists of poorly connected clusters, it may take a longer time for the disease to spread from one cluster to another, resulting in slower disease progression than in a well-mixed population. In some cases, a disease with $R_0 > 1$, which would typically lead to an epidemic in an unstructured model, could become extinct in a network with a small number of initially infected individuals that are not well connected. However, if it enters a cluster, it can spread rapidly within it.

Heterogeneity in the connectivity of individuals can also lead to super-spreaders; well-connected people who become infected can significantly increase the spread^36,42,43^. Also influential is the measure of how well the network is mixed; in assortative mixed networks, individuals of a certain type are more likely to be connected to similar individuals^44^. The likelihood that individuals with similar characteristics or behaviours are more likely to be connected to each other than to those who are dissimilar is called spatial correlation^24,25,45^. For example, spatial correlation is high when heavy drinkers are more likely to be connected to other heavy drinkers than would be expected if the network were randomly mixed. As a result, spatial correlation can affect the spread of behaviours or traits within a network, as individuals may be influenced by their social connections to adopt similar behaviours or traits.

## Methods

### AMHa model on a network

We model the drinking behaviour of individuals as a three-state process, where individuals can be classified as ‘abstaining’ (A), ‘moderate’ (M), or ‘heavy’ (H) drinkers^26^. In addition to regular transitions between states, we also account for superinfections where ‘abstaining’ individuals transition immediately to ‘heavy’ drinkers, and vice versa. Each transition can occur as a result of spontaneous changes or social influence, thus each transition has four rates: $\alpha$ represents the spontaneous transition rate, while $\beta ^A$, $\beta ^M$ and $\beta ^H$ represent the social transition rate induced by abstainers, moderate drinkers and heavy drinkers respectively.

When applied to a non-structured population, this model results in the following system of equations:

$$\begin{aligned} A + M + H&= N\\ \frac{\partial {A}}{\partial {t}}&= - (A \rightarrow M) - (A \rightarrow H ) + (M \rightarrow A) + (H \rightarrow A)\\ \frac{\partial {M}}{\partial {t}}&= - (M \rightarrow A) - (M \rightarrow H ) + (A \rightarrow M) + (H \rightarrow M)\\ \frac{\partial {H}}{\partial {t}}&= - (H \rightarrow A) - (H \rightarrow M )+ (A \rightarrow H) + (M \rightarrow H) \end{aligned}$$

Each of these transitions then occurs depending on an ‘automatic’, or ‘spontaneous’ rate $\alpha$, and, the size of all populations and their corresponding ‘social’ transition rates $\beta$:

$$\begin{aligned} A \rightarrow M&= A(\alpha _{AM} + \beta _{AM}^{M} M + \beta _{AM}^H H) \\ M \rightarrow H&= M(\alpha _{MH} + \beta _{MH}^{A} A + \beta _{MH}^H H) \\ H \rightarrow A&= H(\alpha _{HA} + \beta _{HA}^{M} M + \beta _{HA}^A A) \end{aligned}$$

Other transitions follow a similar pattern.

Note that the epidemiological approach to social contagion does not consider the reinforcing effects of individuals in the same drinking state; it only accounts for the increased likelihood resulting from associations with different drinking behaviours. Although this can be seen as a limitation of the model, including these reinforcing effects would not only increase the complexity of the model, but also exacerbate the limitations imposed by the data set. This is because the social reinforcing effects are disproportionately affected by the sparseness of the social connections in our data, especially in the ‘Friends’ category. This is exacerbated by the high degree of clustering.

Using an approach similar to^24,25^, we can rephrase the Markovian description above as a time-continuous reaction-diffusion process^28,46^. In this interpretation, transitions of each individual belonging to a certain state occur according to a set of interaction rules, described by stochiometric equations. In continuous-time, each transition occurs as a consequence of a set of reaction rates, or, over a small time interval $\Delta t$, a set of transition probabilities. This approach is valid if $\Delta t$ is substantially smaller than the average time to transmission. These probabilities depend on the local network structure of each individual. For example, the transition probabilities for an abstaining individual over a $\Delta t$ time period are:

$$\begin{aligned} P(A \rightarrow M; \Delta t)&= (\alpha _{AM} + \beta _{AM}^{M} N_M + \beta _{AM}^{H} N_H) \Delta t \\ P(A \rightarrow H; \Delta t)&= (\alpha _{AH} + \beta _{AH}^{M} N_M + \beta _{AH}^{H} N_H) \Delta t \\ P(A \rightarrow A; \Delta t)&= 1 - P(A \rightarrow M, \Delta t) - P(A \rightarrow H, \Delta t)\\ P(A \rightarrow A, \Delta t)&= 1 - (\alpha _{AM} + \beta _{AM}^M N_M + \beta _{AM}^H N_H) \Delta t - (\alpha _{AH} + \beta _{AH}^M N_M + \beta _{AH}^H N_H) \Delta t \end{aligned}$$

Analogous mathematical expressions can be derived for probabilities of other state transitions. Note that this model does not incorporate birth and mortality dynamics, as the significance of individual-level network connections in these structured models greatly outweighs the effects of population turnover on spreading dynamics.

### Source data

To validate and calibrate our assumptions and model, we use data from the Framingham Heart Study^47,48^, a longitudinal study of subjects from the town of Framingham, Massachusetts. We used data from both the Original Cohort and the Offspring Cohort during the period 1971 to 2001. The original cohort was examined approximately every 2 years. The Offspring Cohort was examined approximately every 4 years. Both physical and mental health were assessed, as well as behavioural data such as sleep patterns, cigarette smoking and alcohol consumption in the form of self-reported total drinks per week. In addition, a social network was constructed by^13^, based on direct reporting of social relationships by the subjects and other data such as family and address records. This social network includes family members, spouses, friends, co-workers, residential neighbours, and more. In this study we exclude co-workers and neighbours as they have been shown not to influence the alcohol consumption of their connections^15^. In addition, although the type of connection is identified, we simplify the social network by assuming that all connections are bidirectional and that a connection actually exists.

We confirm that all methods were carried out in accordance with relevant guidelines and regulations, as outlined in the ‘Data Use Certification Agreement’, which can be found on the NCBI dbGaP webpages listed in section 5. Informed consent was obtained from all subjects or their legal guardian(s), and we were granted access to all consent groups. All experimental protocols were approved by the Ethical Committee of the Psychology Department at the University of Amsterdam.

### Data processing

In order to fit the AMHa model to the Framingham Heart Study data, a number of data processing steps were required. First, we extracted the self-reported number of drinks for both the original and offspring cohorts for each wave by combining the data for the questions: ‘How many beers/wine/cocktails did you drink per week in the past year’. We then matched the original cohort data to the closest dates of the offspring cohort, resulting in regular intervals between examinations of approximately $\Delta t = 3 \pm 1 \text { year}$. We then restricted the social network to individuals with known drinking data and an age above 21, removing edges between contacts that were shown by^15^ not to be actual social contacts or to actually influence drinking behaviour within this dataset, such as co-workers and geographically close ‘neighbours’.

Additionally, we operate under the assumption that self-reported friendships are reciprocal. This is supported by previous research indicating that within the FHS data, the social influence of perceived friendships falls within a similar margin of error^15^. Given their status as ancillary rather than primary factors in the FHS data, and an average of 0.7 friends per individual, we postulate that underreporting is a more important limiting aspect than directionality. This assumption does not affect the qualitative results of the study. To classify alcohol consumption in different states, we combined information on gender with the number of drinks per week. Then, by comparing the drinking state of each individual in wave *Y* with wave $Y+1$, drinking state transitions were identified. Finally, by integrating this information with the number of connections each individual had in each state, we were able to run the weighted linear regressions that produced the AMHa model parameters.

**Table 1:** Descriptive data of each FHS examination.

Wave	Midpoint year	Age	Egos total	Drinks per day	Contacts total	Contacts who abstain (%)	Contacts drinking heavy (%)
1	1972	47.51	7219	1.07	3.48	0.68 (20)	0.78 (22)
2	1981	53.22	5256	1.00	3.10	0.97 (31)	0.66 (21)
3	1985	54.99	4653	0.90	2.91	1.02 (35)	0.54 (19)
4	1989	57.34	4514	0.76	2.88	1.09 (38)	0.43 (15)
5	1993	59.52	4002	0.72	2.76	1.04 (38)	0.39 (14)
6	1997	58.61	2815	0.72	2.20	0.89 (40)	0.32 (15)
7	2000	61.32	2904	0.77	2.18	0.78 (36)	0.35 (16)
Mean		54.80	4480	0.88	2.92	0.91 (32)	0.54 (18)

Values of age, drinks per day and numbers of contacts are the averages. Only individuals with known drinking behaviour are included. Total contacts contains only individuals whose drinking behaviour is known.

Table 1 provides descriptive statistics for the data used. Note that, apart from the first wave, the average age is 50 to 60 and remains relatively constant over the 20-year period as older individuals from the original cohort die and individuals from the subsequent cohort become older. The data shows a decline in the total number of participants and the average number of contacts with known drinking data over time, as participants pass away without new ones joining. A significant decrease in the prevalence of heavy drinking was observed after the first wave. At the same time, there was an increase in the number of abstainers, ostensibly reflecting a societal shift towards a lower overall prevalence of drinking.

In addition, we analysed the degree distribution (see Supplementary materials Fig. S3 online) and found that it did not vary significantly with different drinking behaviour. Our results show a linearly decreasing degree distribution when considering only those individuals for whom drinking data is available, whereas considering all connections shows an increased variance in degree and a more heavy-tailed distribution.

### Model assumptions validation

When applying our epidemiological model to a network, assumptions are made that need to be validated with our dataset. These are: (1) whether our data are of sufficient quality to serve as a calibration for the model, (2) whether the three-state model proposed by^26^ is an improved representation of real-world dynamics based on the data, and (3) whether our data confirm that the conventional cut-off used to distinguish moderate from heavy drinking based on its biophysiological effects is also applicable to behavioural dynamics.

#### Stability of drinking states

To confirm that the data captures the transitions of each state, it is necessary to show that individuals change drinking states on a timescale of several years, since the time-continuous reaction-diffusion process description, where transitions are described in terms of probabilities, requires that the $\Delta t$ of observations be significantly less than the mean transition time.

In addition, with examinations every 2–4 years, if drinking states were to change annually or monthly, we would be missing information about the dynamics in our data: drinking states in a previous examination would not be predictive of the next, and our observation would not be representative of the individual’s state during that time. We therefore test for correlation between states for all waves and obtain a strong and positive correlation between each individual’s drinking state, with a correlation coefficient *r* ranging from 0.52 to 0.70 per wave, with an average of 0.63. This suggests that current drinking status is a strong predictor of future drinking behaviour and therefore fluctuates not on a timescale of months but on a timescale of several years. It also suggests that all drinking states can be considered stable.

#### Three-state system

In this section we examine whether the use of a three-state system, as proposed by^26^, is supported by our data and is able to capture intricacies that might be missed by a two-state system.

To confirm the latter point, in addition to the correlation results mentioned above, we apply a methodology similar to^26^, where we measure the probability of an individual remaining in the same state over the years and examine the transition rates. The results can be seen in table 2. It shows that the probability of an individual remaining in the same state over several examinations is $75\%$, $67\%$ and $61\%$ for the abstainer, moderate drinker and heavy drinker states respectively. Assuming that the number of individuals who switch between two measurements is negligible, as indicated by the high correlation found, this would indicate a yearly stability of $93.8\%$, $91.8\%$ and $90.3\%$ respectively. Table 2 also shows that there is a small but significant population that transitions directly from heavy drinking to abstinence, a common occurrence when individuals with a drinking problem decide to quit their habit. Next, it shows that moderate drinking acts as a gateway: there are hardly any transitions from abstinence directly to heavy drinking.

**Table 2:** Transmission proportions between different alcohol-use states. Each row sums to one, as it includes all transitions from each state..

From $\backslash$ to	Abstain	Moderate	Heavy
Abstain	0.75	0.24	0.01
Moderate	0.22	0.67	0.10
Heavy	0.07	0.33	0.61

We also look at clustering and spatial correlations. The spatial correlation $C_{XY}$ is the ratio of the observed number of connections between individuals in state *X* to state *Y* compared to the expected number of connections if all states were equal^28^. If $X=Y$, we call this clustering. These measures show whether, for example, abstainers are on average more connected to other abstainers than to heavy drinkers. This clustering could be driven by the spread of behaviour, but could also be attributed to homophily or confounding factors.

Table 3 illustrates the spatial correlations and clustering averaged over all examinations and shows that abstainers and heavy drinkers tend to cluster strongly, being about 1.5 times more likely to be associated with similarly drinking individuals than would be expected. Moderate drinkers tend to have a less strong preference to associate according to drinking behaviour.

This result is a clear indication that within alcohol consumption we cannot assume that the network is well mixed, as the data show that it is assortative. It is therefore beneficial to consider modelling the spread by constraining the model to a network. In addition, it is an indication that each of these three states behaves differently, suggesting that applying a three-state model rather than a two-state model provides a more accurate representation of the real world spread.

**Table 3:** Spatial correlations $C_{i,j}$ and clustering $C_{i,i}$ of connections, averaged over all examinations.

Connected to	Abstain	Moderate	Heavy
Abstain	1.37		
Moderate	0.85	1.07	
Heavy	0.68	1.00	1.54

While the total number of connections is similar: 2.86 for abstainers, 2.74 for moderate drinkers and 2.76 for heavy drinkers, abstainers and heavy drinkers are about $37\%$ and $54\%$ more likely to be connected to individuals with similar drinking behaviour, while being around $32\%$ less likely to be connected to individuals with opposite drinking behaviours. Similar qualitative results are obtained for different waves.

A chi-squared test of independence was performed, showing that they are dependent, e.g., $\chi ^2(2, N = 2109)=100.3, p <0.001$ for heavy drinkers at wave 7. Results for abstainers too were found to be dependent, while moderate drinking was found to be independent.

**Figure 1:**
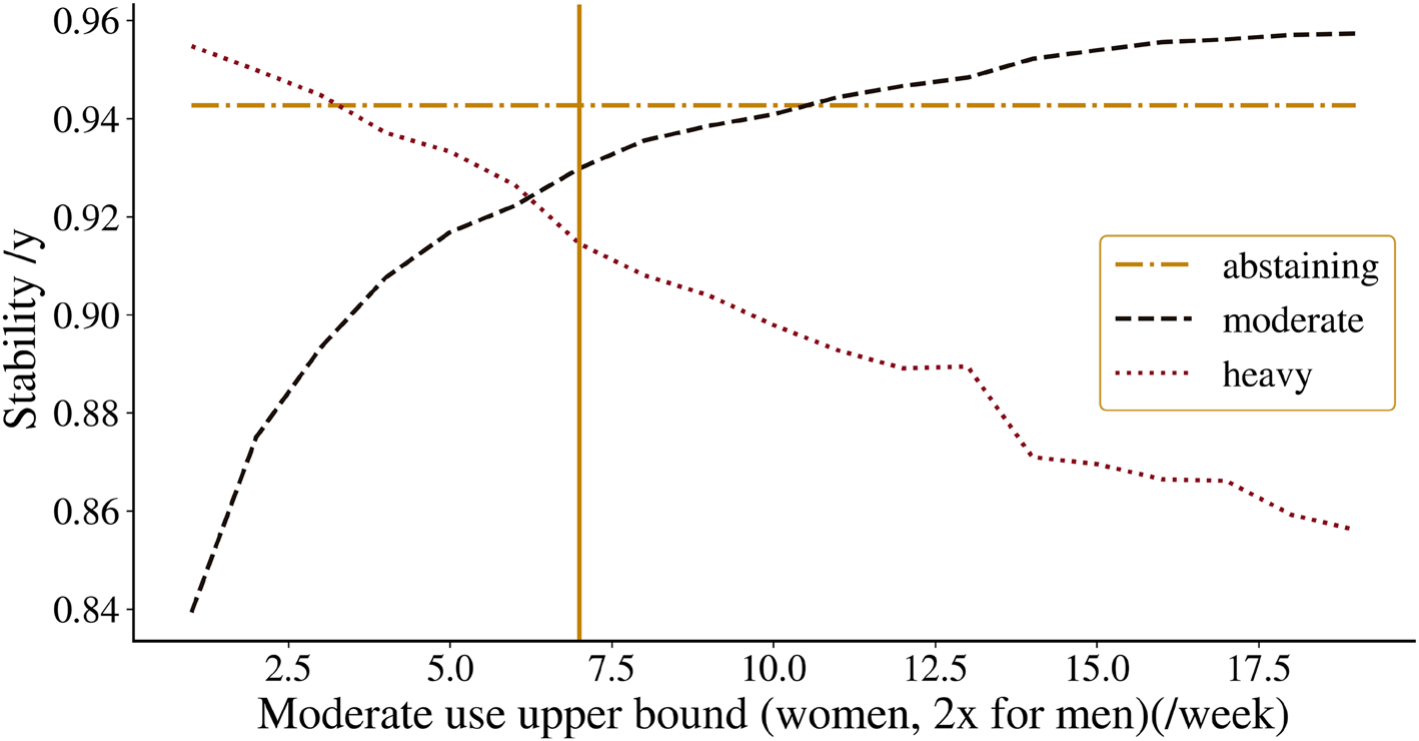
Stability analysis of drinking states across different moderate-to-heavy drinking cutoff values. Shown are the year-over-year stability probabilities for remaining within the same drinking category, across a range of cutoff values used to distinguish moderate from heavy drinking states. The y-axis quantifies the annual stability likelihood, while the x-axis denotes varying threshold levels for the number of drinks defining the moderate-to-heavy transition for women; these thresholds are doubled when applied to men. The vertical dashed line represents the NIAAA consumption cutoff of 7/14 drinks per week for women and men respectively^31^. It can be seen that the overall stability of the heavy and moderate states combined is notably close to its maximum value, supporting the validity of the biophysiological cutoff in the behavioural dynamics of the FHS data.

The cutoff between moderate and heavy drinking, set by the National Institute on Alcohol Abuse and Alcoholism (NIAAA) at 7 drinks per week for women and 14 drinks per week for men^31^, is based on the biophysiological consequences of alcohol consumption and its impact on an individual’s health and well-being. We assess the effect of different cutoffs on the stability of moderate and heavy drinking in Fig. 1. This figure shows the changes in stability observed across all states when different cutoffs for moderate to heavy drinking are applied. Notably, our findings highlight a close match between the biophysiological threshold and the most stable cutoff, as the data show that transitioning between states is most challenging near the biophysiological definition of the cutoff. This suggests that the identified cutoff is not only appropriate from a biophysiological perspective, but is also consistent with the behavioural dynamics observed in the Framingham Heart Study, providing further support that moderate drinking is a distinct state and a relevant addition to modelling efforts.

### Model calibration

To test if and which transitions are socially induced, which are spontaneous and which are a combination of the two, we fit the parameters in the reaction process description of section “AMHa model on a network” for each state. This is done by finding the correlation of all possible transitions with the number of connections an individual has in state Y. A significant positive correlation then indicates that this transition is a socially contagious process. Our methodology, similar to that used in^24,25^, involves running a regression analysis on the transition rate from state X to state Y as function of the number of connections in state Y. Since each individual’s transition is binary, logistic regression is most appropriate. We have performed a comparative analysis (see Supplementary materials Fig. S4 online) which reveals that our logistic regression results are in the linear range, and statistical results closely match. As the number of connections increases, some divergence is observed. However, since the degree distribution is predominantly low, the instances where this divergence is significant are minimal. For example, out of 11,000 observations in the moderate to heavy transition with heavy connections, only 100 instances exhibit this discrepancy. Given that we never encounter rates that exceeds one or fall below zero—because both the slope and the maximum number of connections are low, we conclude that linear regression can serve as a close approximation to logistic regression, and that the epidemiological and social contagion methodology can be applied.

We implement a weighted least squares linear regression, similar to the one applied by^25^. We define the spontaneous transition parameter $\alpha _{XY}$ as the rate at which individuals move from state X to state Y, irrespective of the number of connections in state Y; this is represented by the intercept of the correlation with the number of connections. The social transition parameter $\beta _{XY}^Y$, on the other hand, signifies the increase in the transition rate for each additional connection in state Y, represented by the slope of the regression line. The transition from state X to state Y can also be influenced by connections in state Z, at a rate given by $\beta _{XY}^Z$. Especially when examining the correlation of the transition from abstinent to moderate drinking with the connections of heavy drinkers, as well as the correlation of the transition from heavy to moderate drinking with the connections of abstainers, we expect this to be relevant. We therefore also test for these correlations and, if significant, include them as an additional social term in the model. Lastly, in modelling infectious processes, it is generally assumed that infection and recovery rates remain constant over time. However, given that our data span 30 years, it is possible to examine whether these parameters exhibit temporal variation. Any such variation may indicate significant cultural shifts, such as a change in attitudes towards abstinence, and should be taken into account in the calibration and simulations.

## Results

### Calibrated AMHa model: parameter insights and outcome analysis

We first investigate the trends of infection and recovery rates during our observation period. By comparing the parameter values obtained by a regression on each examination wave separately, we can observe general, long-term trends. Our regression results on the the spontaneous transition parameter $\alpha _{XY}$ and the social contagion parameter $\beta _{XY}$ are presented in Supplemental materials in Figs. S1 and S2 online. They show that all spontaneous rates are statistically significant for each wave and remain relatively constant over time. This is not the case for the socially contagious rates; while there are no apparent trends over time, some rates are not significant, indicating that the data show that their respective transitions are not significantly socially transmitted.

**Figure 2:**
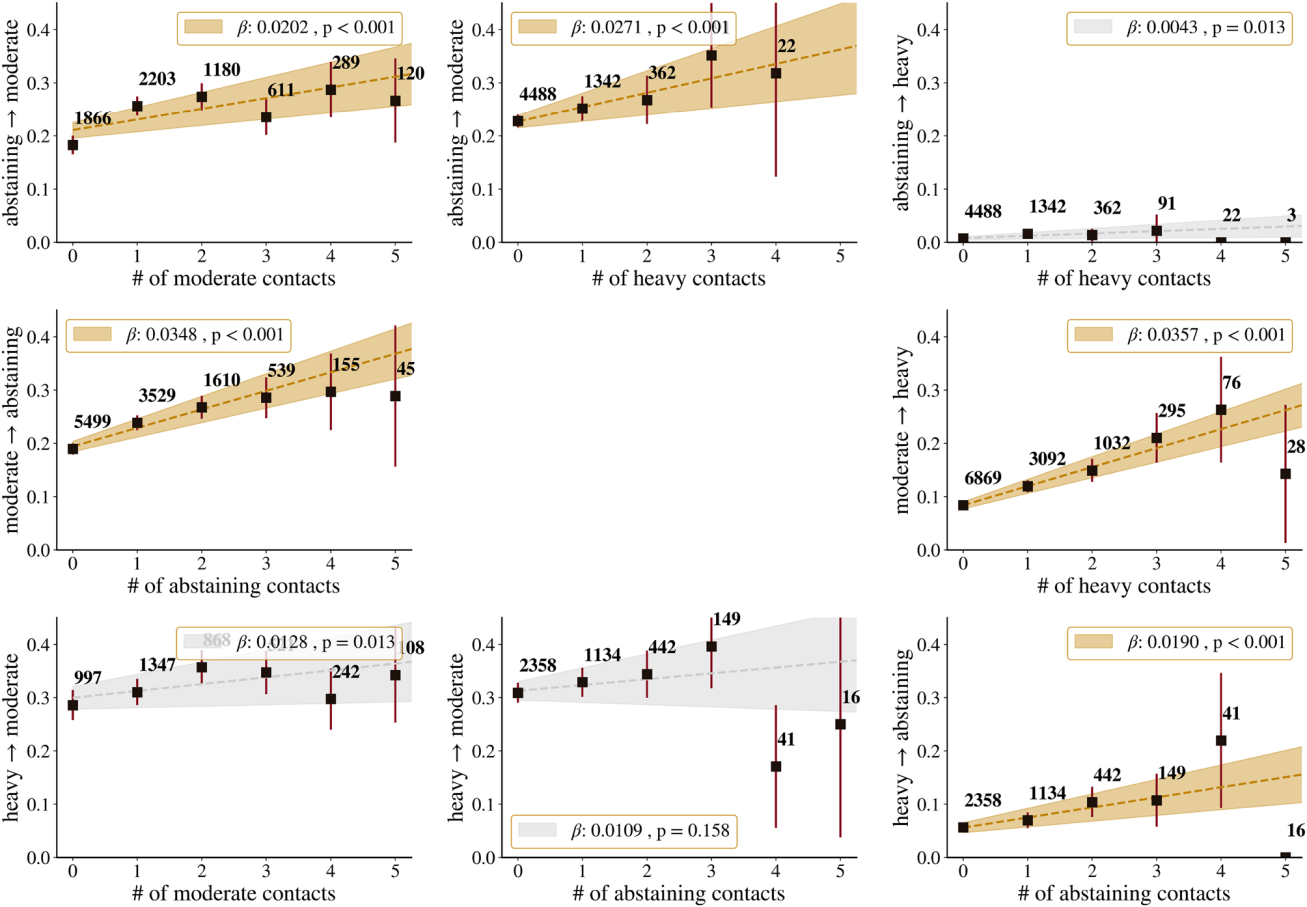
The effect of having multiple social connections in a certain drinking state on an individual’s likelihood to change their drinking behaviour. Shown are the transition probabilities of an individual in state *X* to transition to state *Y* as a function of the number of contacts in state *Y* or state *Z*. Data aggregated over all waves are shown in red, with the number of contacts in state *Y* or *Z* indicated. The results of the linear weighted regression are shown, with the resulting rate and its statistical significance. This regression is shown in grey if no statistically significant slope is found.

**Figure 3:**
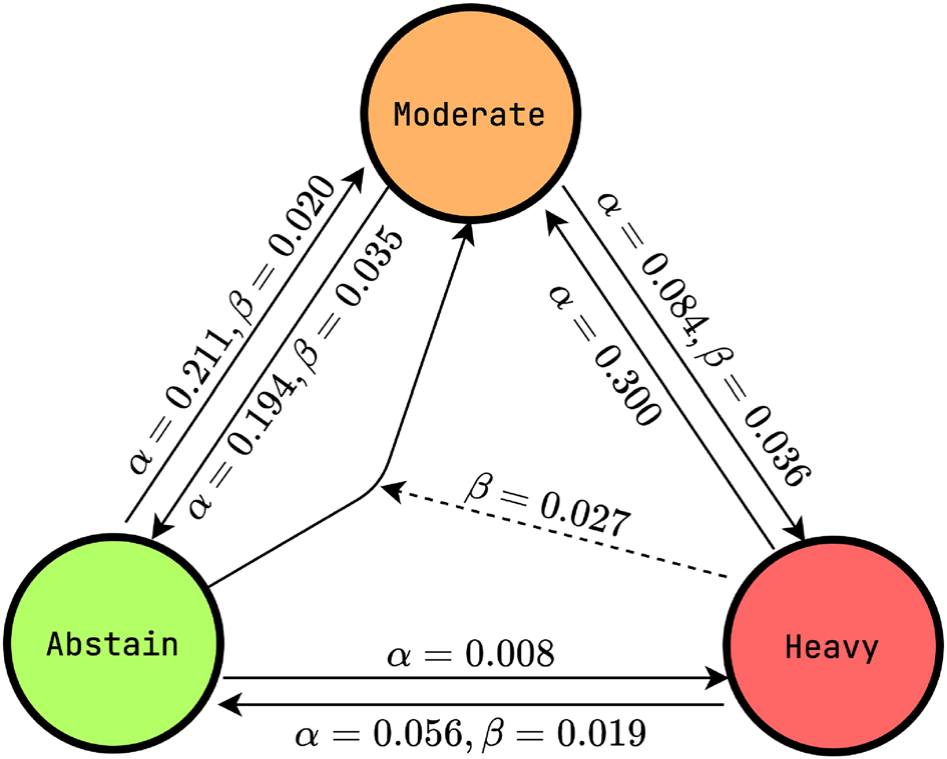
Spontaneous $\alpha$ and social $\beta$ transition rates found for the AMHa model shown in a diagram. All rates shown have $p \le 0.05$. When no $\beta$ is given for a transition, no significant social transmission rate is found. It also shows the effect of heavy drinkers on abstainers to start drinking. These transition rates are the probability that a person in state X will transition to state Y in the next examination period, calculated per 4 years.

As there are no upward or downward trends in the rates across all waves, we follow^25^ and aggregate the data to obtain the regression shown in Fig. 2. This figure shows for all transitions $X\rightarrow Y$ the aggregated data of the transition probability as a function of the number of connections in state *Y*. It also shows the result of the weighted linear regressions, which are coloured grey when not significant. The resulting calibrated AMHa model is shown in diagram form in Fig. 3. For each possible transition, the spontaneous rate is found and, if statistically significant, the social rate is also listed.

We find that the extremes of abstinence and heavy drinking are the most influential: moderate drinking only influences abstainers to start drinking, while moderate drinkers are significantly influenced by both abstainers and heavy drinkers. Heavy drinkers are influenced only by abstainers, who have a significant positive effect on quitting. However, their transition to moderate drinking is not influenced by the number of moderate drinkers or abstainers. Furthermore, abstainers are significantly more likely to start moderate drinking if they have more heavy drinking contacts.

Finally, there is a strong protective effect for extremes: abstainers are more likely to remain abstainers if they have many abstaining relatives. A similar effect is found for heavy drinking. As the default transition is to stay in the same state, this is accounted for by the value of the spontaneous transition rate.

### Simulations

In this section, we analyse the calibrated AMHa model to predict future prevalence rates for heavy drinking and abstinence. Additionally, we evaluate interventions and formulate hypotheses regarding the efficacy of potential policies. To conduct simulations, we use the network at the time of the third exam, which strikes a balance between decreasing network density over exams while being representative of future networks based on model parameter fits.

**Figure 4:**
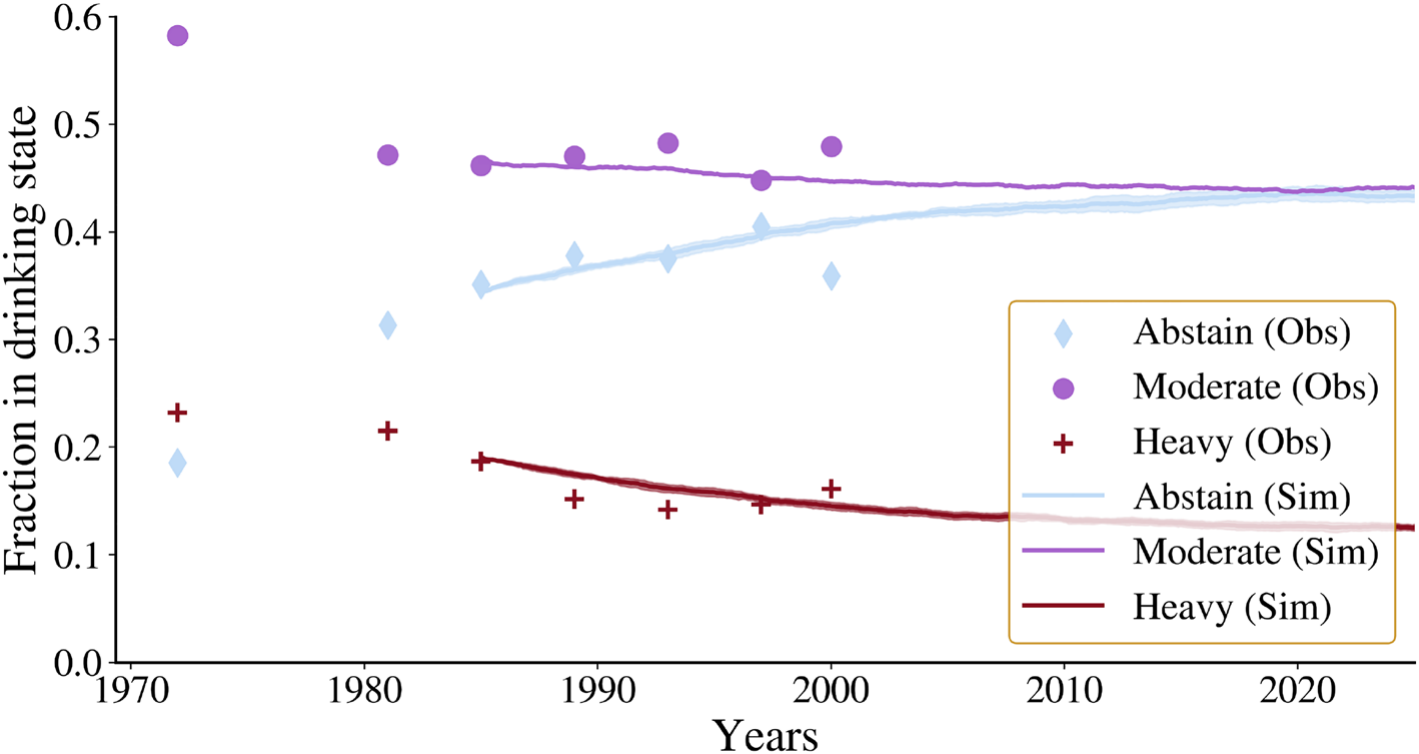
This figure displays the evolution of drinking behaviour fractions over time for both the observed and simulated network. The points represent the fractions observed in the examination data, using the midpoint of the examination duration. On the other hand, the simulated fractions are based on the third observation’s data and network, forecasting future fractions using obtained model parameters while remaining within the wave 3 network.

Figure 4 shows the population-level proportions of drinking states from the model and the observed statistics in the FHS data. The divergence of our individual-level model fit from the population-level results can be attributed to several sources. For the sake of consistency in our analysis, we use the midpoint of the examination period as the observation date, even though the examinations took place over several years. Therefore, there may be differences in the estimates due to associated errors or due to parameter variation between examinations. However, we have shown that the regressions for the model parameters fall within a similar range (see Supplementary Figs. S1 and S2 online). Thus, differences between the model fit and the study data are primarily due to the dynamic network: the social network of the observed data changes over time, whereas the simulations are run on a static network. Despite the different fitting methods and the sources of variation mentioned above, we can see that the general trend is reasonably consistent with the population level data. Our simulations suggest that the future prevalence of heavy drinking will decline and stabilise at 14% by 2025, while abstinence rates will increase and stabilise at around 43%.

#### Simulating interventions

**Figure 5:**
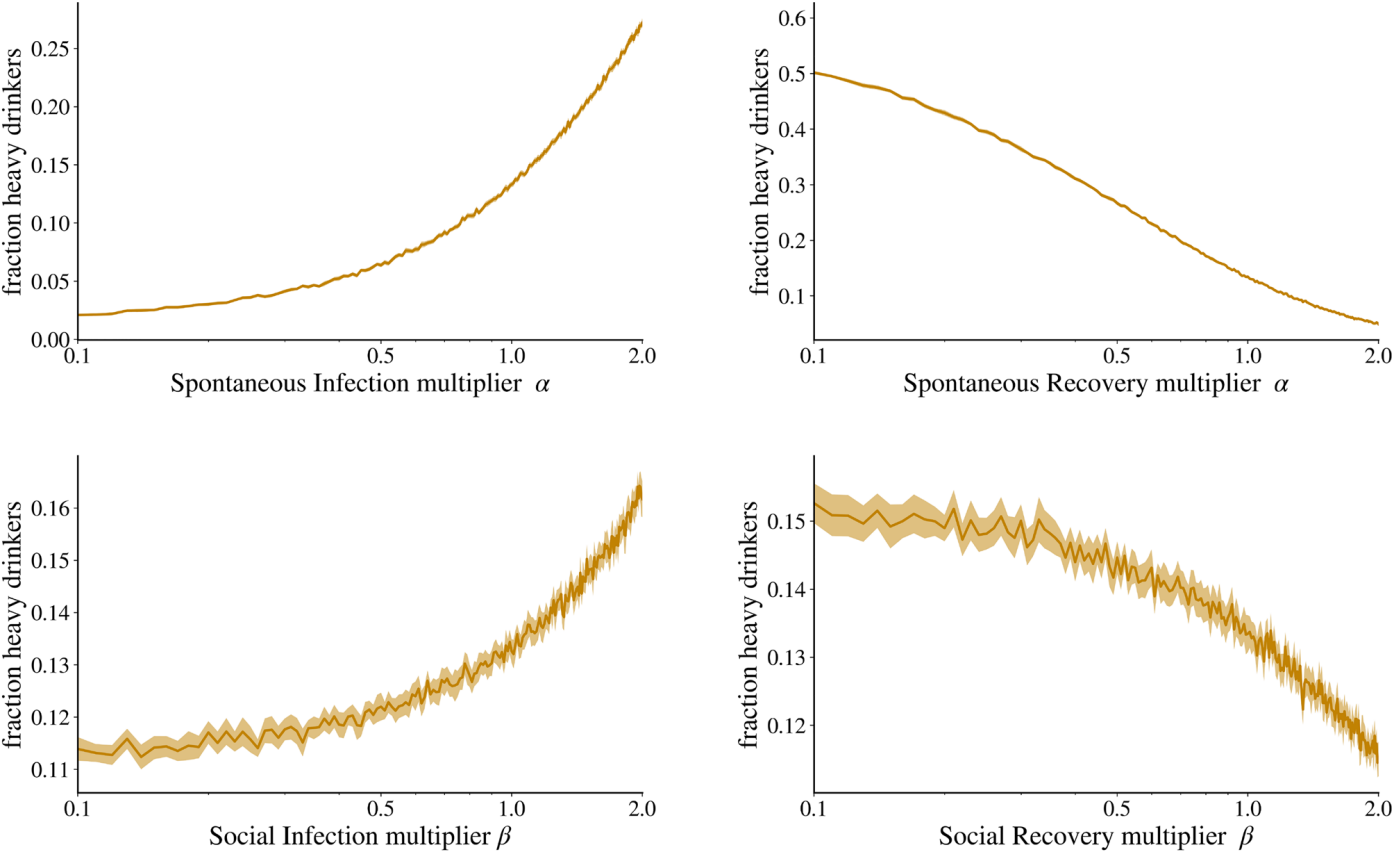
This figure shows how changes in ‘positive’ and ‘negative’ social influence affect the steady state of heavy drinkers in a stable, endemic state 30 years later. Shown is the average of the stable state proportion of heavy drinkers after 30 years across 33 different studies, including a confidence interval. Note that all rates have been multiplicatively adjusted to maintain their relative proportions. The x-axis uses a logarithmic scale to ensure that a doubling or halving of the rates is equally distant from the base values.

ere, we explore the dynamics of the epidemiological model by comparing the effectiveness of increasing the social influence of connections in a lower drinking state with decreasing the social influence of higher drinking states. Examples of interventions that have this effect could be increasing the social stigma of drinking (increasing the social influence of abstainers^49^) or improving education about the negative effects of drinking (reducing peer pressure to drink^50^). Interventions that increase or decrease the number of abstainers or heavy drinkers in one’s social network have a similar effect on the dynamics, such as joining Alcoholics Anonymous^51,52^. Figure 5 shows the results of changing all the ‘increasing’ versus ‘decreasing’ social influence parameters. We assume that it takes the same amount of effort to increase or decrease these parameters through interventions. We therefore use a logarithmic scale, so that halving is the same distance from the baseline as doubling, and they can be compared. We can see that increasing social recovery rates has an increasing effect, while the spontaneous multiplier has a decreasing effect. Similarly, interventions that reduce infection rates have a decreasing effect.

**Figure 6:**
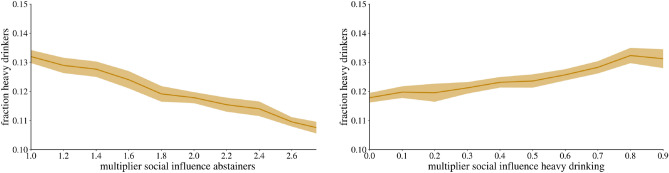
This figure shows how changes in social transmission rates for abstainers and heavy drinkers affect the proportion of heavy drinkers in a stable, endemic state 30 years later. These changes are relative, with increases in transmission from moderate drinkers to abstainers ($M \rightarrow A$) and from heavy drinkers to abstainers ($H \rightarrow A$) of up to almost three times the magnitude. Similarly, the effect of heavy drinking is halved at 0.5 and reduced to a tenth at 0.1.

Next, Fig. 6 compares the effectiveness of policies that either increase the social influence of abstainers $M\rightarrow A_A$ and $H\rightarrow A_A$ or decrease the social influence of heavy drinkers $M \rightarrow H_H$ and $A \rightarrow M_H$. Again, we simulate the network for 30 years, at which point the network structure is stable, and compare the resulting proportion of heavy drinkers in the network. If we compare a doubling and a halving of the social impact of abstainers and heavy drinkers respectively, we see that the fractions are above 12% and below 12%. This suggests that although both have a significant impact, focusing on increasing the social impact of abstainers on their environment may be more effective than reducing the impact of heavy drinkers on their environment. This tendency is reinforced if the policy is more effective, as a multiplication or division by three results in a $10\%$ difference in the proportion of heavy drinkers.

## Discussion

We analysed alcohol use and network data from the Framingham Heart Study and found evidence of social spread of alcohol consumption among connected individuals. Using this data, we have developed an epidemiological model that incorporates three distinct and stable states of alcohol consumption: abstinence, moderate drinking and heavy drinking. It captures the interplay between spontaneous and socially driven drinking behaviours, yielding transmission rates that quantify the impact of social contagion.

When examining the network structure, we find that heavy drinkers and abstainers are significantly more likely to be connected to others with similar drinking habits. Specifically, heavy drinkers and abstainers are $43\%$ and $54\%$ more likely to be associated with similar drinking individuals. This highlights the importance of incorporating network modelling into studies of drinking behaviour, as epidemiological models that assume homogeneity in populations do not accurately capture the complexities of such behaviours. In addition, we found that a three-state categorisation gives rise to states that are all stable, each with distinct infection dynamics, and that the biophysiological threshold of 7 or 14 drinks per week for women and men corresponds well with the stability of these classifications in our data. This threshold can therefore also be considered appropriate for behavioural dynamics.

After fitting our model, we discovered that both abstainers and heavy drinkers have a significant impact on the drinking habits of their social connections, and that this influence remained consistent over the 30-year data period. We found that each abstaining connection increased the probability of a moderate drinker to also abstain by $18\%$, while each heavy drinker increased probability to become heavy drinker by $40\%$. We also observed that abstainers had a significant positive influence on heavy drinkers to quit drinking. Conversely, for each heavy drinking connection of an abstainer, the probability to start drinking and become a moderate drinker is significantly increased. While moderate drinkers were found to have a small but significant impact on encouraging abstainers to start drinking, they had no significant effect on helping heavy drinkers reduce their alcohol consumption.

Based on our findings, we conclude that social-drinking plays a significant role in non-problematic drinking and that abstainers too are not immune to peer pressure. Moreover, increasing alcohol use to the level of heavy drinking is largely influenced by the social environment, but reducing drinking is not, as the spontaneous rate of reduction in drinking occurs for $7.5\%$ of the population each year, regardless of the number of moderate drinking connections. Although transitioning to total abstinence occurs in only $1.8\%$ of the heavy drinking population, being surrounded by abstainers increases the likelihood of achieving total abstinence by almost $50\%$ per connection.

Using this calibrated model, we simulate the future prevalence of abstinence, moderate drinking and heavy drinking. We find that heavy drinking will continue to decrease to around $13\%$, down from $22\%$ in 1975, and abstainers increase to a value very similar to moderate use, of $43\%$. Further, we investigate the general epidemiological dynamics of the AMHa model on the FHS social network. We find that, assuming that efficacy of policies are relatively similar, increasing the social impact of abstainers is more efficient than decreasing the social effect of heavy drinking individuals.

The FHS dataset is unique in that it combines a longitudinal social network with drinking data over a long period of time. In addition, all participants lived in the same city, which means that many social connections are individuals who are also included in the study. However, as obtaining a social network was not an aim of the study, many social connections were recorded indirectly. Therefore, it is not always clear whether the connections obtained from the unnamed data are people with whom the participant is actually in contact. This could lead to inaccuracies in the social network compared to reality. In addition, we apply an undirected network; however, there could be differences in influence depending on the directionality of the connections: parents will be more influential on their children than the other way around. Another limitation of the dataset is its demographic composition, as it consists mainly of older subjects. As a result, the behaviours and interactions we observed reflect this older cohort and may not be representative of the patterns exhibited by younger adults or adolescents.

In addition, this epidemiological model assumes a linear relationship between the probability of spreading and the number of connections; while this holds for our data with a limited degree distribution, larger data may reveal a more complex relationship. Furthermore, our model does not take into account the non-Markovian elements of alcohol consumption behaviour. Recovered former heavy drinkers have a significantly higher risk of returning to their previous behaviour in the long term than those who have never abused alcohol. This poses a challenge in measuring transition rates from abstinence to moderate or heavy drinking, as these rates may differ between individuals at different stages of drinking behaviour. Additionally, a significant fraction of heavy drinkers never attempt to recover and continue to drink heavily for years. Conversely, another group may be actively trying to recover with varying degrees of success, leading to different recovery rates within the heavy drinking population.

Although challenging, future work on this topic should therefore attempt to capture the complex and catastrophic nature of substance abuse^8^. Models integrating psychologically based theories of alcohol use and its impact on the social environment would be able to incorporate non-Markovian dynamics and differentiate between individuals based on their history. Such models require careful development and testing, but hold great potential for deepening our understanding of substance abuse.

These models would benefit greatly from incorporating even larger datasets with more measurement points, both in terms of time and network size. Furthermore, the robustness of the models could be improved by validating them against different datasets, both at the individual level and at the population level. Moreover, although the FHS data already contains some information about the types of social connections, methods that differentiate between the social relevance of each connection (e.g. time spent or influence on each other) could improve the representation of real-world connections. In addition, simulation studies examining the impact of network structure and investigating super-spreaders, different network scenarios, and various spatial-correlation factors could provide a more comprehensive understanding of the effectiveness of adjusting social environments, for which the AMHa model could prove to be a suitable starting point.

### Supplementary Material

Supplementary Information 1.

Supplementary Information 2.

Supplementary Information 3.

Supplementary Information 4.

## Data Availability

The data, comprising clinical exams and demographic details such as age and sex, are sourced from the Framingham Cohort study (reference: phs000007.v33.p14). The social network information is derived from the FHS-Net Social Networks substudy (reference: phs000153.v9.p8). The supporting data for this study’s findings are accessible via the NCBI database dbGaP. However, due to restrictions on these data, which were utilized under license for this investigation, they are not publicly accessible. Contact information, details on the data, and instructions for requesting access can be found on the following websites: Framingham Cohort: https://www.ncbi.nlm.nih.gov/projects/gap/cgi-bin/study.cgi?study_id=phs000007.v33.p14, FHS-Net Social Networks: https://www.ncbi.nlm.nih.gov/projects/gap/cgi-bin/study.cgi?study_id=phs000153.v9.p8).

## Code availability

The source code used in this research is available at^53^. Note that it separates the data wrangling code, which requires access to the data, from the simulations, which only require included calibrated model parameters.
