# Supplementary figures and images for: Alcohol consumption as a socially contagious phenomenon in the Framingham Heart Study social network
Source: Sci Rep. 2024 Feb 24;14:4499. doi: 10.1038/s41598-024-54155-0 (PMC11052543; doi:10.1038/s41598-024-54155-0)

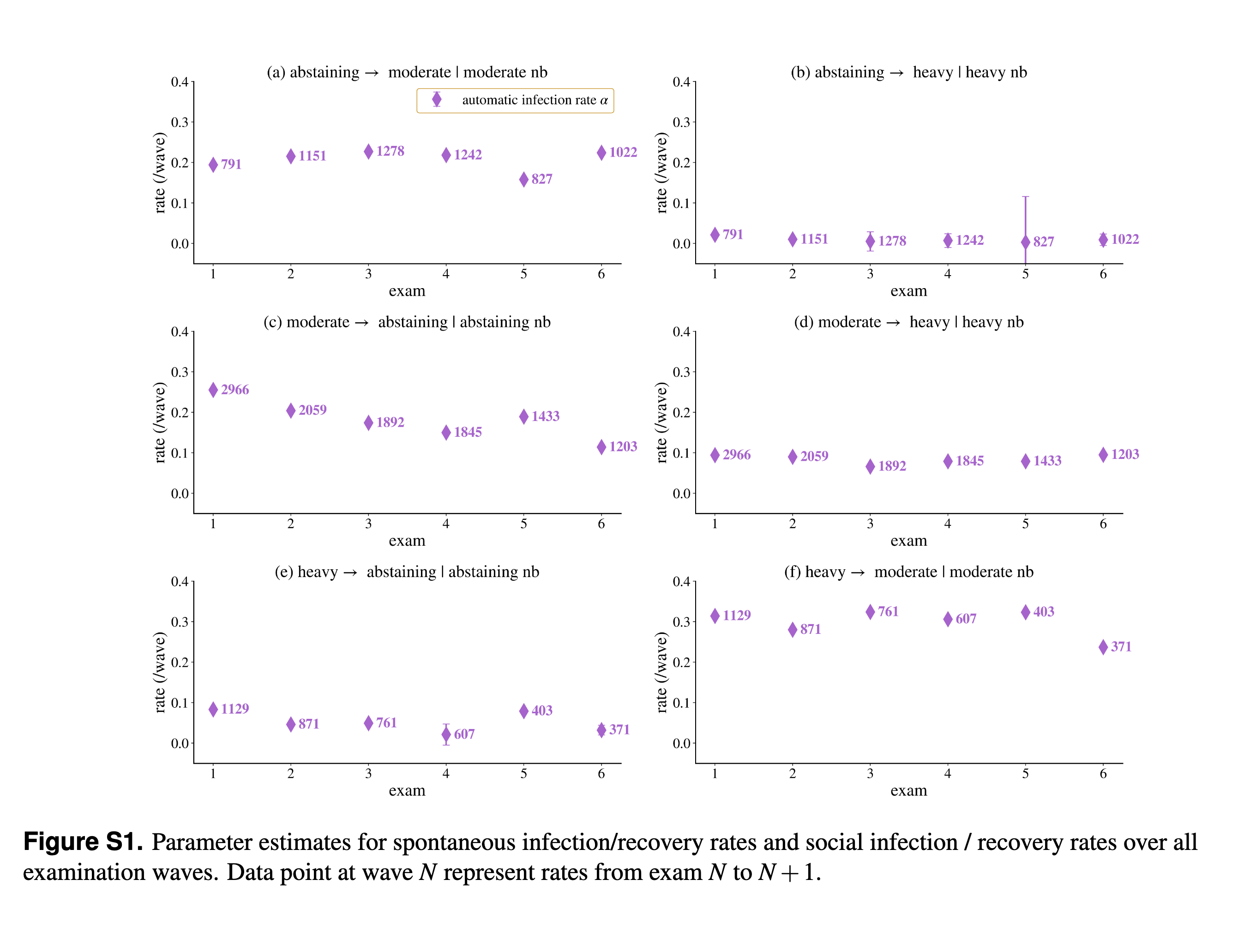

Supplement: Supplementary file 1 — Supplementary Information 1. [file 41598_2024_54155_MOESM1_ESM.png]

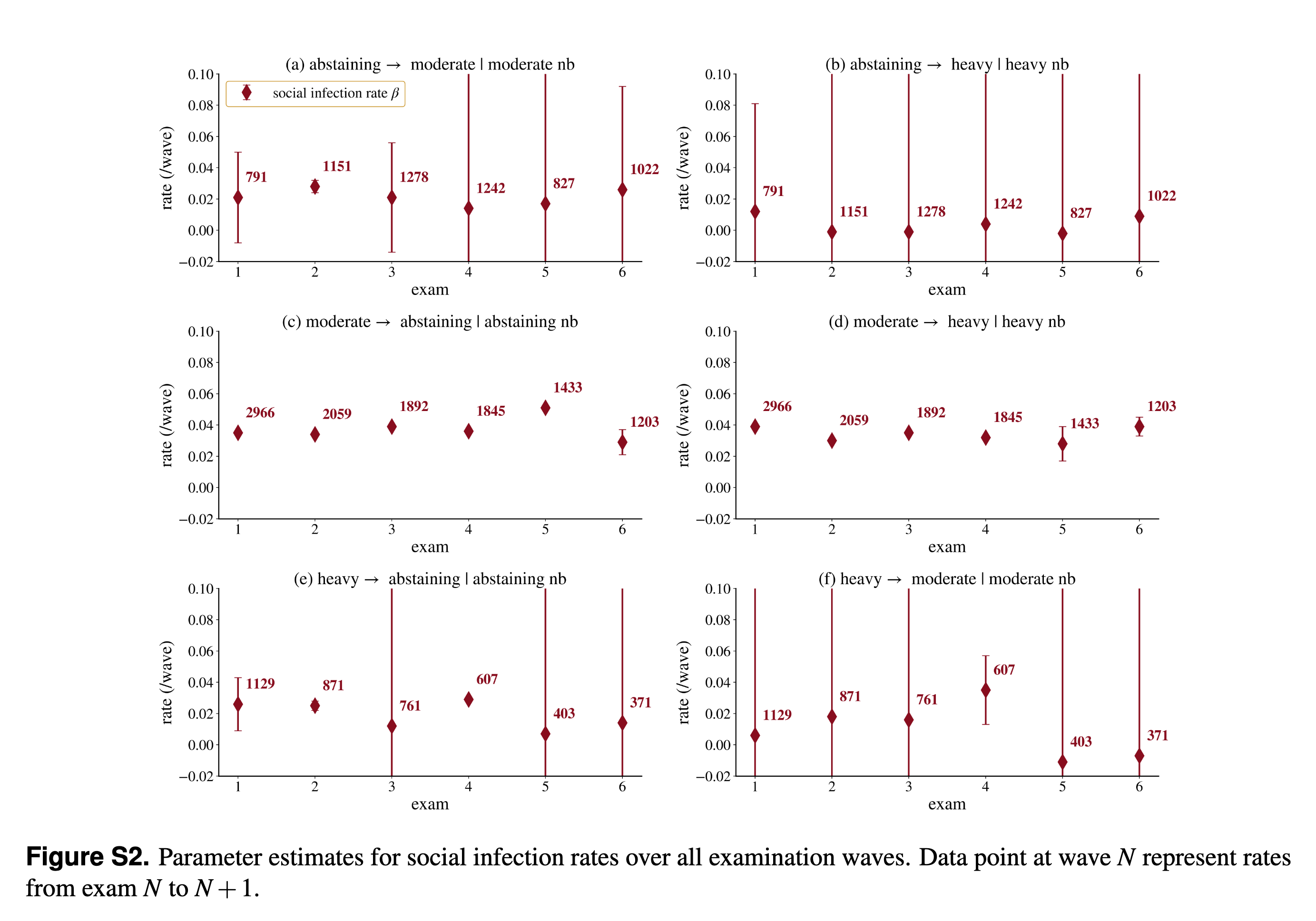

Supplement: Supplementary file 2 — Supplementary Information 2. [file 41598_2024_54155_MOESM2_ESM.png]

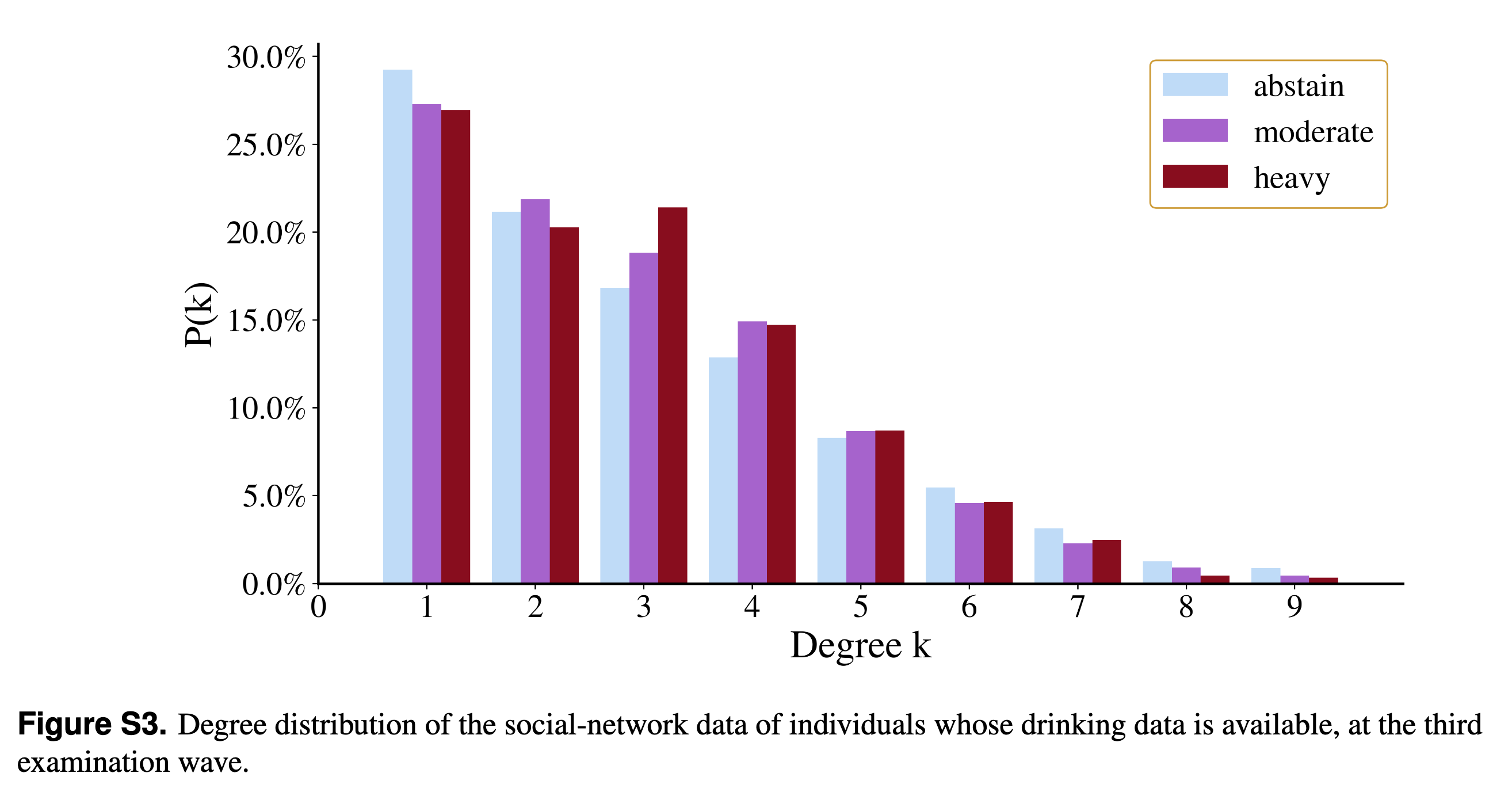

Supplement: Supplementary file 3 — Supplementary Information 3. [file 41598_2024_54155_MOESM3_ESM.png]

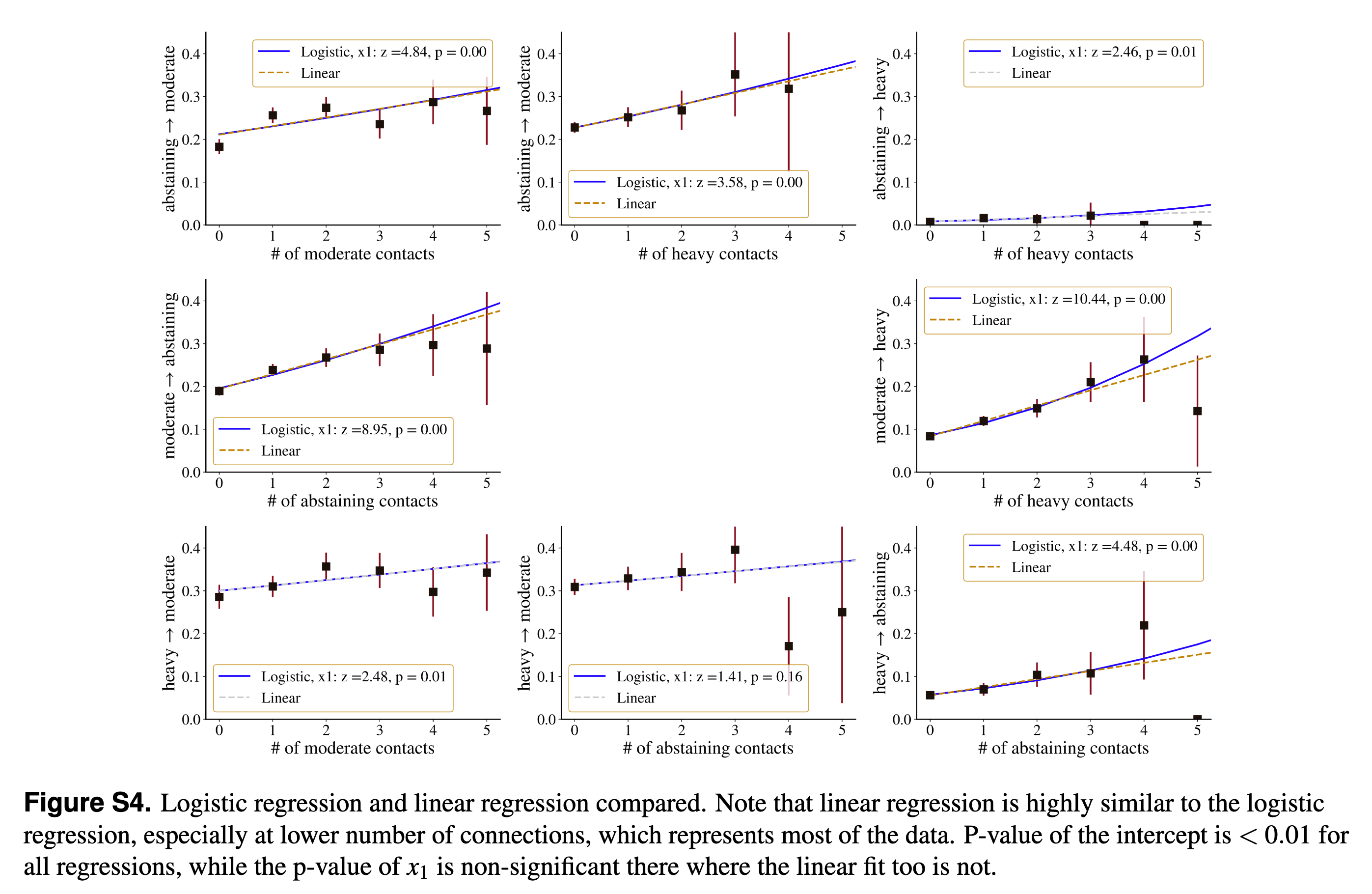

Supplement: Supplementary file 4 — Supplementary Information 4. [file 41598_2024_54155_MOESM4_ESM.png]
